# Adaptations to family-based treatment for Medicaid-insured adolescents with anorexia nervosa

**DOI:** 10.3389/fpsyg.2024.1389652

**Published:** 2024-09-13

**Authors:** Renee Borges, Peyton Crest, John Landsverk, Erin C. Accurso

**Affiliations:** ^1^Department of Health Professions, University of San Francisco, San Francisco, CA, United States; ^2^Rhodes College, Memphis, TN, United States; ^3^Oregon Social Learning Center, Eugene, OR, United States; ^4^Department of Psychiatry and Behavioral Sciences, Philip R. Lee Institute for Health Policy Studies, University of California, San Francisco, San Francisco, San Francisco, CA, United States

**Keywords:** adaptation, family-based treatment, implementation, anorexia nervosa, publicly-funded settings, culture

## Abstract

**Background:**

Family-based treatment (FBT) is the leading intervention for adolescents with anorexia nervosa (AN); however, it is under researched in socioeconomically disadvantaged and racially diverse youth.

**Methods:**

Semi-structured interviews were completed with ten FBT clinicians who practice in publicly-funded settings. Interview questions were focused on implementation challenges, overall acceptability and appropriateness of FBT, and naturally-occurring treatment adaptations.

**Results:**

Content analysis revealed common themes relating to the implementation of FBT in publicly-funded, community-based settings: acceptability and appropriateness, complexity and learnability, perceived core components of FBT, cultural adaptations, socioeconomic factors, logistical considerations, organizational and systemic barriers, training acceptability, participant’s self-efficacy, and telehealth accommodations.

**Conclusion:**

The discussed themes offer insights into the implementation of FBT for settings with limited resources, aligning with prior research on clinical adaptations for multicultural patients. Recognizing these themes can guide clinical adjustments and refine the adapted treatment model in real-world settings for patients facing systemic barriers.

## 1 Introduction

Anorexia nervosa (AN) is a restrictive eating disorder (ED) that carries one of the highest mortality rates of all psychiatric disorders. Onset most commonly presents during adolescence (American Psychiatric Association, 2013; Micali et al., 2013; Petkova et al., 2019) with a lifetime prevalence rate of 4% for females and 0.3% for males (Arcelus et al., 2011; van Eeden et al., 2021). It is characterized by pervasive weight and shape concerns and a distorted body image leading to restricted dietary intake and significantly low weight (American Psychiatric Association, 2013). Prolonged malnutrition can result in chronic medical complications causing an increased risk of death (Peterson and Fuller, 2019). To minimize the impact of medical complications and increase the likelihood of recovery, early intervention for AN is crucial given the higher rate of recovery for adolescents with a shorter duration of illness (Steinhausen, 2002; Jassogne and Zdanowicz, 2018; Neale and Hudson, 2020).

Family-based treatment (FBT) is currently the leading treatment for adolescents with AN (Lock et al., 2010; Lock and Le Grange, 2015; Gorrell et al., 2019) given its strong evidence-base (Agras et al., 2014; Gorrell et al., 2019). FBT emphasizes a key shift in perspective by viewing the patient’s family as a resource for recovery while placing them as active members during the treatment process (Gorrell et al., 2019). This method highlights an agnostic stance towards the development of AN by externalizing the illness (Lock and Le Grange, 2015). In other words, FBT clinicians frame ED behaviors as outside the patient’s control, without placing blame on patients or parents, and work to foster healthy interpersonal relationships (Lock and Le Grange, 2015; Gorrell et al., 2019).

While FBTs efficacy is well-documented in academic medical centers, there is limited research examining FBT outcomes in community-based settings, and even less focused on publicly-funded settings (Le Grange et al., 2015; Accurso et al., 2021). As a result, the effectiveness of FBT is unstudied in Medicaid-funded services, despite the fact that Medicaid is the largest payer for U.S. mental health services (Hayes et al., 2015; Halbeisen et al., 2022). Racial and ethnic minorities make up most of the population who are newly eligible for Medicaid (Hayes et al., 2015), emphasizing the importance of understanding how FBT applies to racially and ethnically diverse youth. Indeed, the demographic profile of youth with EDs is diverse (Marques et al., 2011; Huryk et al., 2021). This is reflected in recent findings suggesting that not only are ED rates leveling across diverse demographic groups, but health-related impacts are the highest among marginalized populations who may have limited access to specialized services (Mitchison et al., 2014). Recent research in Medicaid-insured youth with EDs also highlights the racial and ethnic diversity of this population (Accurso et al., 2021, 2024).

Cultural adaptations to evidence-based treatments enhance the quality of services for historically disadvantaged groups by increasing feasibility and appropriateness that is rooted in cultural context (Griner and Smith, 2006). Culturally adaptive interventions have been shown to significantly improve mental health outcomes (Alegría et al., 2016), which may be particularly important for the highly diverse population of Medicaid recipients. Treatment adaptations within FBT might address specific barriers that Medicaid-insured patients and their families often face, such as inflexible work hours, food insecurity, and misunderstandings or stigma about eating disorders (Lau, 2006; Yu et al., 2019; Accurso et al., 2021). One recent study highlighted several adaptations made in practice to FBT when working with diverse families (e.g., addressing systemic barriers early in treatment) (Dimitropoulos et al., 2024).

However, research on adaptations is nascent and has focused largely on adaptations for specific cultural groups, without examining socioeconomic factors that may impact treatment appropriateness, acceptability, or effectiveness. To advance the science of adaptations for lower-income populations receiving FBT, it is important to examine naturally occurring adaptations employed to address real-world barriers. This study qualitatively examined how community-based clinicians implemented FBT in publicly-funded clinics with Medicaid-insured adolescents with AN, including a review of implementation challenges, treatment acceptability, and adaptations. Mixed methods were also used to examine patient outcomes, including weight restoration as a primary outcome, early treatment response, treatment completion, and clinician assessment of patient response to treatment. This is one of the first descriptions of FBT implementation and outcomes for Medicaid-insured youth, which fills an important gap in the literature that has not traditionally focused on lower-income youth with EDs or those served in low-resourced settings (e.g., publicly-funded clinics). Furthermore, these findings have broader implications beyond FBT for Medicaid-insured families. Insights from this study may be applicable to other manualized protocols for low-income and diverse families, enhancing their applicability and effectiveness in various settings. This can lead to improved mental health outcomes across a range of disorders and treatment modalities, ultimately contributing to the reduction of health disparities and the promotion of equity in mental health care services.

## 2 Materials and methods

### 2.1 Qualitative

Six publicly-funded agencies in San Francisco County that provided intensive services were informed about a training opportunity in FBT given rising recognition of a gap in services for youth with AN in the county. Given their knowledge of the child system of care, leadership within the San Francisco Department of Public Health (SFDPH) were responsible for targeted recruitment of agencies that would best facilitate ultimate implementation. In determining which and how many agencies to target, they balanced factors related to patient demand, provider training needs, geography, language, and knowledge about the extent to which agency leadership would be supportive of a new training initiative. SFDPH wanted to make sure that there was sufficient patient demand for interested clinicians to receive consultation on an active case. Based on their calculation for demand for services, they restricted the number of agencies approached to achieve a training cohort of approximately 20 providers, given that some providers were attending to gain exposure to FBT only and anticipating that others would ultimately choose not to implement the intervention. SFDPH hoped that 6–8 clinicians would decide to implement the intervention, which would meet the demand for services and ensure that interested clinicians would be able to gain experience treating a case with FBT soon after training. They also wanted to minimize patient/family travel for care (i.e., agencies were geographically spread across the county) and prioritized agencies with Spanish language capacity, given that 60% of San Francisco’s ED cases were Latinx and Spanish-speaking. Clinicians (*N* = 25) completed a two-day in-person training and those who were interested in seeing ED cases were invited to join a year-long consultation group. Initially, 11 clinicians expressed interest in the consultation group. Within the first few meetings, one clinician decided to discontinue due to competing obligations. All remaining clinicians (*n* = 10) who participated in the consultation group following the initial FBT training were recruited; all provided informed consent to participate.

A qualitative approach was employed to understand participants’ experiences implementing FBT in publicly-funded settings with Medicaid-insured youth and their families. Semi-structured interviews were conducted by the senior author with each participant following the one-year consultation period post-training. Open-ended interview questions were focused on implementation challenges and facilitators, acceptability and appropriateness of FBT, and naturally occurring treatment adaptations, including rationale and perceived impact on outcome. Additional information on methods is detailed elsewhere (Accurso et al., 2021). All study procedures were approved by the IRB at the University of California, San Francisco.

### 2.2 Case series

De-identified information was collected from clinician participants about their patients with AN, Atypical AN, or other similar restrictive eating disorder. Demographics were collected for patients and caregivers, including age, race, ethnicity, language, and education level. Clinical information was also collected, including primary diagnosis, co-occurring diagnoses, and treatment history.

Clinicians recorded the session number, date, and weight after each session. The patient’s height was taken at the start of treatment. Using the BMI approach, each patient’s median BMI (mBMI) was determined by calculating their BMI relative to the 50th percentile BMI for their exact age and height as indicated on the CDC BMI-for-age percentiles chart. A BMI at the 50th percentile signifies the expected median value within a cohort of typically developing adolescents, serving as a reference for %mBMI.

### 2.3 Data analysis

A content analysis was used to interpret qualitative data. This approach relied on a lower level of inference interpretation, with researchers focusing on explicit descriptions of content (Vaismoradi et al., 2016). The interviews were recorded, transcribed verbatim, and reviewed several times by the research team. This coding framework was developed using an inductive coding method, allowing themes to naturally emerge from the data through systematic analysis and team consensus. Several themes emerged after initial review of the transcripts, giving rise to a coding framework that was implemented to capture content and significant themes. To improve inter-rater reliability, the coding team, consisting of two researchers (RB, PC), independently applied the framework to the transcripts, followed by discussions to resolve discrepancies and refine the coding scheme. Disagreements in code application with particular text excerpts were resolved through consensus-making meetings, including a third researcher (EA) to resolve discrepancies as needed. Revisions to the coding structure were made as the interviews were analyzed. Qualitative data was analyzed and interpreted using the *Dedoose* software. *Dedoose* was chosen due to its user-friendly interface and capacity to collaborate in real-time via a cloud-based platform that allowed for blinded double-coding. Summative content analysis was used to quantify` the frequency of each code and how the clinicians describe similar experiences regarding the feasibility and adaptability of implementing FBT in publicly-funded settings (Hsieh and Shannon, 2005). Each code was contextualized by identifying patterns and application frequency throughout the ten interview transcripts (Vaismoradi et al., 2016).

## 3 Results

Ten clinicians participated in this study from six different sites. Participants were mostly women (90%, 10% male) with a mean age of 31.9 years (*SD* = 3.21; range: [27,37]). Participants identified their racial and ethnic identity as non-Hispanic White (*n* = 4), Latinx (*n* = 3), Asian (*n* = 2), and Black (*n* = 1). Clinician participants also reported on cases that they treated with FBT. Demographic characteristics for patients (*N* = 6) in the case series are presented in Table 1. Qualitative themes are presented below in order of highest to lowest frequency.

**Table 1:** Demographic characteristics of adolescent patients and their caregivers.

Patient	1	2	3	4	5	6
Age	16	16	14	13	17	15
Gender identity	Cisgender female	Cisgender female	Cisgender male	Cisgender male	Cisgender female	Cisgender male
Race/Ethnicity	Asian	Mexican	Central American	Asian		Mexican
Language(s)	English, Nepali	English, Spanish	English	English, Cantonese	English, Arabic (comfortable)	English, Spanish (comfortable)
*Caregiver 1*						
Relationship status	Married	Married	Single	Married	Separated	Married
Employment	Nanny	Restaurant	Warehouse employee	Homemaker		
Education	High school	Some high school	High school	High school		
Race/Ethnicity	Asian	Mexican	Central American	Asian		Mexican
Language(s)	Nepali, English (not comfortable)	Spanish, English (not comfortable)	English	Cantonese, English (not comfortable)	Arabic, English (not comfortable)	Spanish (somewhat comfortable)
*Caregiver 2*						
Relationship status	Married	Married		Married	Separated	Married
Employment	Uber driver	Chef at a restaurant		Grocery store owner		Construction
Education	High school	Some high school				
Race/Ethnicity	Asian	Mexican		Asian		Mexican
Language(s)	Nepali, English (somewhat comfortable)	Spanish, English (somewhat comfortable)		Cantonese, English (not comfortable)		Spanish, English (somewhat comfortable)

### 3.1 Intervention characteristics

The most frequent themes were related to the overall treatment principles of FBT as well as clinicians’ experiences implementing the treatment in their respective settings. Subthemes for this section include *acceptability and appropriateness, complexity and learnability, and perceived core components/family involvement.*

#### 3.1.1 Acceptability and appropriateness

Clinicians frequently referred to the relative advantages of using FBT in their clinical settings. Therapists reported appreciating that the FBT framework is consistent with a strengths-based treatment approach. Additionally, they welcomed the treatment’s emphasis on family empowerment and acknowledged the benefits of staying symptom focused. They also made references to the effectiveness of FBT and its suitability to the circumstances and presentations of patients within their practice. For instance, one clinician expressed:

“It really does support the family in feeling less overwhelmed by something that is really scary for them. It is focused on eating behaviors, and it does help in recovery for young people.”

Indeed, the outcomes observed in the case series underscore positive trends in the engagement of adolescents undergoing FBT for AN. Three of the six patients restored weight to the median (100% mBMI). The remaining patients were above 100% mBMI at treatment onset, and further weight restoration or modest weight gain was clinically appropriate. Of these, one gained weight and the other two maintained their weight over time. The two adolescents who discontinued FBT reached 100% mBMI well before termination.

Therapists also emphasized the acceptability of FBT in accommodating various family dynamics. They highlighted its flexibility in involving all family members and aligning treatment expectations with the family’s available resources. Another clinician stated:

“Off the bat I thought ‘Well, I think actually this will be pretty easy to implement,’ because it does come from this ‘The family is the expert’ plan, and it comes from this natural ‘You should be eating what your family is eating,’ which really sets itself up for the cultural humility perspective, and in some ways makes it easier to learn about your patients, because you're learning about their food cultural habits.”

#### 3.1.2 Complexity and learnability

Clinicians discussed how they were able to grasp FBT training material and subsequently implement the treatment in their respective settings. Many clinicians reported that the treatment was comprehensible and well-defined. For example, one clinician noted:

“As a model, it’s one of the more learnable ones. It is clear and builds on the basic skills you need. I have seen lots of different people learn it and do well in learning it, so that was encouraging. I also observed in the case that I had briefly, and in the cases that I saw other people have for longer term, that people became increasingly comfortable, and I felt increasingly comfortable. It’s a very learnable, applicable model.”

Another clinician reported:

“It was really easy to learn. However, it was not necessarily easy to master.”

Several participants attributed the ease of understanding FBT to its manualized style, with structure and resources provided during the initial training. Specifically, they expressed that they relied on session outlines for Phase I (i.e., one of several resources created by the trainers to support implementation) but struggled with changing treatment material to appropriately adhere to the needs of certain families. Clinicians frequently noted high training acceptability, but as they treated patients with intersecting psychosocial stressors, they were challenged to consider ways in which they might make nuanced shifts in typical protocols or treatment goals to better suit their patient population. While clinicians were able to understand the treatment framework, they endorsed some difficulties applying this framework to diverse families with unique situations, exemplified by the following quote:

“It was not complicated to learn the basics, but I think as we learned over time, a lot of the nuances of practice do take time to incorporate, and families all look different. So that’s why I love having the ongoing consult group, because as we continue to get new clients, we’re going to have different questions. Because I feel like there’s a greater array in how our clients present compared to the manual. Those all looked more or less the same, we get a lot more diversity.”

#### 3.1.3 Perceived core components and caregiver/family involvement

Participants commented on core components and treatment principles within FBT. One participant noted the importance of weight restoration for psychological recovery:

“… we say in the beginning, ‘All these things are wrong—you feel depressed, you feel anxious, your trauma is bothering you, but you’re also not eating. And once we get you eating, we hope that all these [other] things will decrease as well.’ Once she became re-nourished, it was like she was a whole different kid.”

Another participant highlighted the importance of family and caregiver involvement as a core component:

“I would say [that] attendance of family members [is core to FBT]. Being rigid on waiting for family members. … And having everybody hold responsibility. Having the power dynamic change is core but also … empowering for the parents.”

Several participants also expressed how family involvement as a core principle contributed to therapeutic collaboration in reaching treatment goals. However, therapists discussed how treatment was complicated when financial and other systemic barriers interfered with caregivers’ availability and treatment engagement. Clinicians acknowledged the importance of caregivers holding responsibility for treatment progress, while also expressing concerns around the feasibility of this model for some families who might not be able to consistently engage in treatment or be available to monitor meals/snacks at home. Therapists discussed how lower levels of family engagement did not necessarily equate to a lack of parental commitment to or endorsement of FBT. Rather, they highlighted how cultural and socioeconomic factors often constricted caregivers’ ability to fully engage in treatment in the ways the FBT model traditionally demands.

### 3.2 Adaptations and flexibility

Clinicians frequently discussed FBT’s flexibility in adapting the protocol in response to various barriers to better fit their patient’s unique needs. Subthemes for this section include c*ultural, socioeconomic,* and *logistical considerations.*

#### 3.2.1 Cultural considerations

This theme encompassed adaptations made in response to cultural barriers that affected treatment implementation. Participants frequently discussed how differences in religious traditions, foods, family dynamics, and language with multicultural patients often required additional knowledge and special consideration. Most patients in the case series were bilingual with parents whose primary language was not English, presenting some language barriers in treatment. These adaptations are not necessarily distinct from adaptations made in the context of implementing FBT in other settings. However, the number and frequency of adaptations required to appropriately tailor the intervention to the diversity of families served in publicly-funded settings was quite high. One example of a cultural consideration is described in the following excerpt about one clinician’s experience working with a Middle Eastern family approaching Ramadan:

“Ramadan was starting soon, and it was expected that she would fast… but I was like ‘Oh, it’s not going to work.’ … I had to go do some research about Ramadan because I didn’t want to make them have to teach me everything about their culture… A lot of those things came up that altered how I would've gone in with my session agenda and what I would do.”

Several clinicians also noted how mental health stigma in certain cultural groups may have impacted treatment engagement. One participant highlighted:

“There was so much stigma around them coming in for treatment that I was so focused on them not dropping out of treatment. There was a lot of mental health stigma that interfered with participation.”

Some clinicians noted that spending extra time discussing the core components of FBT and the importance of family as agents for recovery was critical to increase caregiver understanding, buy-in, and willingness to engage in treatment. They also discussed the importance of setting expectations for family involvement during the first sessions. Participants frequently addressed the need to emphasize the concept of *externalizing the illness* when working with multicultural families to avoid placing blame and judgment on family members. When maintenance in recovery would have been aided by change within the family system, this became more challenging, as illustrated by the following quote:

“It was hard for me to address intergenerational patterns of eating—the parents knowing that it’s their eating patterns too, and their views. Setting more expectations of maybe it’s the system and maybe it’s not just the youth… I struggled later on bringing it up because then it seems blaming.”

Clinicians shared the effects of racial and ethnic discrimination on the family system. They also emphasized how cultural beliefs about EDs were important factors to incorporate into sessions. One therapist cited how minority stress often influences a family’s interpersonal dynamics and can be attended to during FBT sessions. This concept was illustrated here by one clinician:

“Gender roles have a stronger generational effect in a lot of marginalized populations or communities… transgenerational trauma can be so immense. The family that I worked with had a lot of political and other persecution throughout their lives, so it just made their ideas a lot more rigid and stuck about their child, and it increased the guilt of the child around their eating disorder.”

Another clinician discussed the impact that immigration status, language capacity, and role reversal within family dynamics has during FBT sessions:

“In this case, you had a real role reversal in the family because you had a parent who was an immigrant and a child who was born in the United States, and because of that, there'd been a role reversal in the family, where sometimes the child was more of the navigator of the world who helped the parent to understand things. And so FBT, saying to mom, ‘You're the one in charge. You're the one in power,’ was really not how they functioned and didn't really acknowledge the ways in which mom needed the kid to navigate certain things, and also the ways in which the kid had this power or control that mom couldn't stop…. The client's level of school-based education was far beyond mom's already at this point, and so saying, ‘You're the parent, and you know best,’ wasn't the way they'd operated.”

This quote highlights how the FBT approach, which places the parents in charge of treatment, was in misalignment with longstanding parental and child roles that may be present in immigrant families, particularly for caregivers with limited English proficiency.

#### 3.2.2 Socioeconomic considerations

Clinicians commented on their initial concerns that FBT might not be an appropriate intervention for Medicaid recipients. In the case series of six patients, all families were from a low socioeconomic background, with parents functioning as essential workers. Clinicians discussed subsequent adaptations they made during FBT to more appropriately address socioeconomic factors in treatment, with several participants noting trepidation about implementing FBT with this population:

“I was a little bit worried about how we could adapt this model that requires a lot of resources and flexibility… It’s really hard to get parents involved in treatment. We may have one [parent] but not the other. So initially, I was a bit concerned at how the rigidity of FBT was going to fit into our clients, who need a lot of flexibility.”

Another challenge involved addressing eating disorder behaviors before attending to other issues, which was emphasized in training as a core component of FBT. Several clinicians highlighted how holding off on addressing other concerns is often unrealistic for families who face significant socioeconomic challenges that impede their ability to stay symptom-focused, especially when other concerns require urgent attention (e.g., school refusal impacting caregivers’ capacity to work and earn money to support their family). One clinician underlined the innate privilege one would need to hold in order to solely focus on eating disorder symptoms:

“Addressing just the eating disorder [without attending to other issues] is a privilege, and our families don’t have that privilege. I think of myself as a privileged person coming to my patients and saying, ‘Don’t think about that; we’re only thinking about eating disorder issues.’ It's not sensitive and can be detrimental.”

Several therapists attributed their hesitancy towards implementing FBT in community-based settings to concerns regarding financial insecurity. This concept was also illustrated in the following quotes:

“How do you give your kid excess calories when you’re struggling to even put any food on the table?”

“It wasn’t challenging to follow the structure. But it was challenging in the context of my client’s life. There’s a lot of lack of financial resources, lack of access to food, lack of access to comfortable housing …, which added another layer of how complicated it became for her to just focus on the eating disorder.”

In order to address food insecurity and lack of financial resources, one clinician discussed how they adapted the treatment plan to include additional case management sessions:

“With my family, they didn’t have money to buy food. So I had to adapt a lot to talking about how we’re going to get meals in the first place. I had to be very validating. I didn’t want to put the family in a situation where I would shame them for not having the resources … I’m not a case management person. I had to adapt to the family’s needs in the context of case management, spending time for collateral work with Mom–doing research about food banks and other nonprofits that can offer food. I wrote a letter for housing so they could get a bigger place.”

The clinicians reflected on the struggle to balance the framework of FBT while accommodating the practical limitations resulting from socioeconomic disparities such as fewer resources and time availability. One clinician highlighted various challenges related to socioeconomic stressors for one of their patients:

“So [caregivers attending all treatment sessions] is the frame I come in with, and I also recognize that for various reasons that’s not always the case. As there are families who would love to and want to, and the mom has two jobs. There’s nothing I can do about that… I try to think, who are the people who can participate much more frequently? Who can we pull in if it’s not the two parents? What does a household look like? Who can come in? … Sometimes it has to be modified times [for appointments], so that in two-caregiver households, they can participate. I think that’s hard. Because I’m like, ‘If these families were upper middle-class, and maybe worked nine to four, didn’t have these other barriers or challenges to access to care, can afford to pay out of pocket, or had extended family or resources, like people resources to watch the other kids if needed, I’m sure they would do it.’ And so trying to think about that in a way where it is prescriptive, and how do I stay within the frame [of FBT], and work around the availability of the family?”

#### 3.2.3 Logistical considerations

Participants acknowledged barriers to treatment implementation pertaining to difficulties adhering to logistical and operational aspects of FBT with publicly-insured patients and their families. In addition to scheduling difficulties, many therapists noted transportation to the clinic as another important logistical barrier to treatment engagement. One participant stated:

“A lot of our families don’t have cars. A lot of our families don’t have transportation… I was worried about picking up these clients to bring them back to our office. That’s something that we have the capability of doing, but then, is that going to impact my role and how they see me?”

To mitigate these concerns, many clinicians used other mediums to conduct sessions if the families were not able to attend in-person sessions, as expressed by one therapist:

“We’ve been using Zoom more, and in the Medi-Cal world, I see this as an opportunity where parents on their lunch break can attend virtually. One parent can step in for half the session, the other parent for the other half.”

One clinician shared a case example where she wanted the patient’s older sister to participate in sessions. However, this clinician adapted the structure of sessions since the sister’s work schedule was a barrier to attending an hour-long weekly session:

“It didn’t make sense for her to miss two to three weeks and then show up one week…. It wasn’t helpful. Her presence would then throw things off because she would have so many questions about things that we already had discussed. I adapted the sessions [so that] the sister was involved at the end of each session. We would have take-home points that the patient would share with her sister about what we talked about. Then I would ask mom to also talk to the sister as well.”

Several clinicians cited the impracticality of involving family members in weekly sessions. In some cases, clinicians accommodate logistical concerns by implementing a separated FBT model. Several clinicians grappled with how to stay within an FBT framework to the extent possible, while also recognizing the constraints of what the family could realistically manage, as explained by one clinician:

“If the parent is not involved, then it’s not really FBT…. It would be possible to work with the client and the parents completely separately and then come together once in a while, but I also don’t know if that would be FBT. That's a pretty big modification, but I think it can be effective if the barriers don't get in the way, just like any therapy. If the socioeconomic, the scheduling, the language barriers—all these things don't get in the way of a couple of the core concepts, I think it can be helpful.”

### 3.3 Organizational setting and systematic barriers

There were several comments aimed at internal and external contextual factors that influence the implementation of FBT.

#### 3.3.1 Inner setting

Most participants discussed how internal organizational factors often influenced treatment implementation. These factors included the use of an interpreter, collaboration with the multidisciplinary team, standardized assessment protocols, care coordination, and treatment planning.

Clinicians noted the challenges of working with a high acuity population in a community mental health setting that lacked an interdisciplinary team, presenting numerous obstacles to effective treatment. This construct was used to capture information regarding the social architecture, age, maturity, size, or physical layout of the organization. This theme also encompasses references to inner setting factors that affected the implementation of FBT. Several clinicians discussed the importance of coordination with the medical team. For example, one clinician noted:

“There is a thin link between the medical and mental health team. I wonder about warm hand-offs because so many families were told to come to us by the medical team, and the urgency faded by the time they came to us.”

This clinician went on to emphasize how *“shared understanding of goals”* between the medical and mental health team is imperative for treatment outcomes. Another clinician stated:

“One of the biggest challenges was trying to coordinate care with the pediatrician who was not at UCSF on the specialized eating disorder team, and she often said things to the family that ended up undermining the goals that we were working on… It was much harder working with a physician who wasn’t an ED expert and who didn’t really value my expertise.”

Within the inner setting, clinicians made remarks about the need for increased collaboration with medical providers to ensure alignment on treatment goals. They also expressed how increased collaboration also contributed to a sense of stability and security for families because they would consistently hear the same key messages across different members of their care team. This concept is illustrated here:

“Increasing medical collaboration with the medical team would be great. Looking back, something that could have been helpful for me would have been having a meeting with my patient’s doctor, like a face-to-face meeting.”

#### 3.3.2 Outer setting

Several participants discussed factors outside of the clinic, agency, or organization that influenced the treatment process. These factors may include external partnerships, the county or Department of Public Health (DPH), and providers at other organizations within the county.

A few clinicians spoke about navigating communication channels with schools and higher levels of care, as exemplified by one clinician’s statement:

“Residential treatment is not funded by DPH. When we see that a kid needs residential, we don’t have the power to say, ‘Okay we’re moving them up to residential.’ We have to go to these other systems and convince them that this kid needs a higher level of treatment…. They will only do that if the issue is impacting their education.”

### 3.4 Less frequent themes

#### 3.4.1 Training acceptability and consultation

Clinicians shared their perspectives about the relevance of the two-day FBT training and year-long consultation group with regards to real-world implementation. In general, all participants spoke highly about the initial training and consultation groups, regardless of their previous experience working with ED patients. Most clinicians noted that having experienced FBT clinicians available for consultation and learning about clinical cases during the consultation group was a valuable resource for becoming more comfortable with real-world implementation.

When asked about ways to improve the appropriateness of the training for providers working with Medicaid-insured youth, some participants noted that aspects of the FBT manual were less relevant for their patient population. Many participants noted how the FBT manual included examples of families of privileged socioeconomic classes and dominant cultural identities. They expressed how these examples offered less applicable information to the population they were serving. One participant explained:

“I read the FBT manual, and it was definitely not made for our population…. Some of the things that families would do, like vacations or brunch or horses or whatever. It was not conducive to our population or didn’t feel relatable. I was hesitant and wary because of that. When reading the book, it catered more to a family that might have more resources.”

When asked about other areas of focus that should be included in the training, another clinician suggested:

“High calorie, nutrition information. Something that is accessible to people that we can support them in adding to their foods. I remember in one of the training sessions, there was the explanation of the stomach and how much room like chicken takes and how much room oil takes. And it’s the same amount of calories. I thought that was super helpful to conceptualize. Also how to support clients and families in brainstorming what to add to the meal.”

#### 3.4.2 Clinician characteristics

The participants were asked questions regarding their previous clinical experience before becoming involved in FBT and how they perceived their self-efficacy while learning and implementing the treatment in their settings. In general, the clinicians were apprehensive about beginning to implement FBT, in part due to concerns about FBT’s appropriateness for their patient population. Many participants specified how the shift in therapeutic stance was difficult. For example, one clinician recounted a time she was tasked to explain the core components of FBT to a family in a grave manner:

“‘Terrible things will happen if you don’t do this’. It’s a different approach, and I think it was one that was harder to transition into for myself.”

#### 3.4.3 Telehealth accommodations

Participants discussed the impact the COVID-19 pandemic had on treatment implementation during the shelter-in-place order. They discussed how many of their patient’s families faced increased socioeconomic stressors during the pandemic. However, many clinicians noted the benefits of incorporating telehealth in FBT sessions to mitigate logistical barriers. One clinician stated:

“I want to make it an option again, with an adjusted schedule virtually, and if there’s a way we can continue to offer that for families who don’t have to take three, four buses just to get there. Granted, we could do a hybrid model, in person once a month or twice just to kind of have eyes and check in, but really offer [telehealth] as a way of increasing access and making it more flexible to those families that, again, don’t have to go across town or don’t have- it’s a whole host of things. It makes it easier in some cases.”

#### 3.4.4 Co-occurring themes

**Figure 1:**
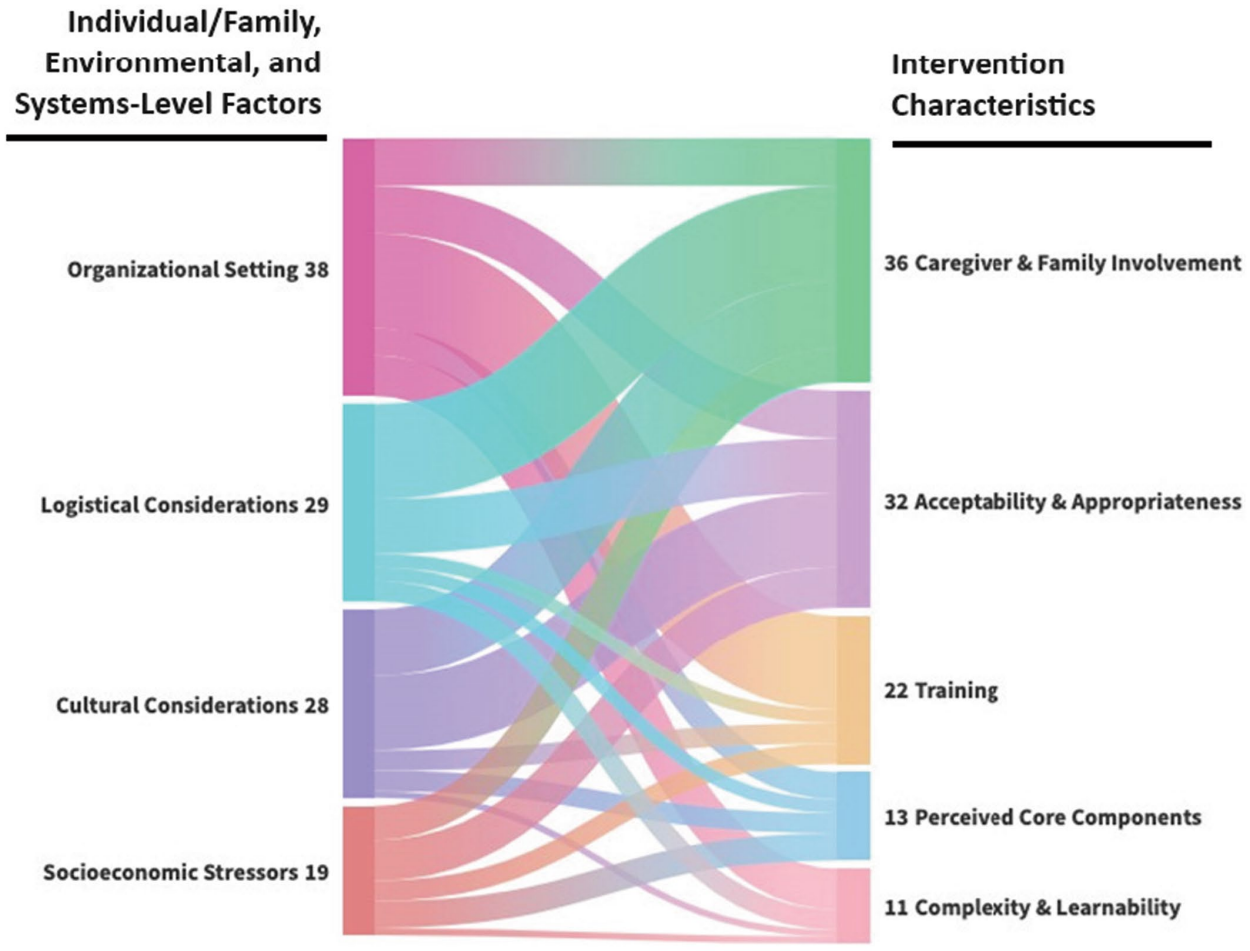
Alluvial chart of co-occurring code applications linking individual/family, environmental, and systems-level factors to intervention characteristics.

Figure 1 maps the relationship between intervention characteristics and individual/family, environmental, and systems-level factors (which may represent implementation barriers and/or facilitators). Each factor in the alluvial chart flows into its corresponding intervention characteristic with stream’s width being proportional to the frequency of code co-occurrence. The most frequent co-occurrence illustrates how organizational setting characteristics (Column 1) impacted the training needed to support implementation (Column 2), while logistical and cultural considerations (Column 1) had the biggest influence on caregiver and family involvement in treatment (Column 2). In contrast, the acceptability and appropriateness of FBT (Column 2) was impacted relatively evenly by organizational setting, logistical considerations, cultural considerations, and socioeconomic stressors (Column 1). Table 2 describes suggested intervention adaptations and strategies for addressing implementation barriers.

**Table 2:** Implementation barriers and suggested interventions.

Theme	Example quote
*Cultural adaptations*	
Communicating about lack of progress without disempowering or discouraging caregivers.	“And then [caregivers] get frustrated, like ‘We’ve been doing everything, but they are still not moving forward.’ …I feel frustrated as well, and asking families, what do they think? And if we are really stuck, if I suggest something, how would it land? Because we really want families to come up with their own ideas from a strength-based perspective…. Because I remember a couple times in interactions, when a *señora* told me, ‘*No lo hice bien* [I did not do it right].’ And I was like, ‘Wait a minute. Uh-oh. Now she feels like she’s doing something wrong.’ And then I should shift whatever messaging, or how it is I’m delivering this information or this intervention to the parent because she’s feeling, ‘I’m not doing something right’ or ‘*Estoy fallando* [I’m failing],’ versus let us try something different and thinking in a more collaborative way.”
Maintaining an agnostic view of the illness while simultaneously being able to address maintaining factors (e.g., disordered beliefs about food/weight or eating within the family)	“But then if you bring [family eating patterns] up later on, it’s kind of framing it as, ‘Oh the problem is what’s grandma eating….’ When if it would’ve been built in the framework earlier—intergenerational patterns of eating—and parents knowing that it’s their eating patterns too, and their views…. And setting more expectations of maybe it’s the system and maybe it’s not just the youth. How is this youth’s problem perpetuated without it being caused by anything… Because I struggled later on, bringing it up once I started to conceptualize that, because then it seems blaming—not having that as a framework from the beginning made that go slower.”
Treatment considerations when working with a family with high levels of criticism	“I thought I was going to have to go to a separated model…. I thought partially because of the family’s schedule and partially because their interpersonal dynamics were really very critical, and from a cultural perspective—the family was critical, but also the hierarchy within their own individual culture was very critical. I definitely think a cornerstone [of FBT] is placing no blame, and really understanding that the child is doing the best they can: ‘This is an eating disorder taking over them.’ At the beginning I was like, ‘I do not know that I’m ever going to get these parents onboard, and I think that they are going to be critical throughout all of treatment, and we are going to have to move to a separated model.’”
*Training*	
Include examples of patient and families with intersecting identities and increased stressors and how clinicians navigate implementation barriers	“Bring in examples relevant to the kinds of cases that they see of thinking of reducing challenges and barriers and building on a family strength and strength base so that there is not a lack of engagement—we do not lose a family because they feel misunderstood or misheard. I think back to those core tenets of, ‘They want to get better. They love and care for their child.’ And this is true of FBT too, right? I guess it’s how it’s presented. The family looks very different, and there’s a family or natural supports building on that. Who’s the community that we can tap into? And empowering people who feel marginalized to be able to help their child.”
Discussions around treatment flexibility and adaptability	“When we think of families who have a lot of psychosocial stressors, certain things were presented in a way that they could not be flexible, or not that they could *not* be flexible, but there needs to be some standard of practice in a set frame, which I understand. Though, I also understand that there has to be some sort of flexibility to attend to the psychosocial factors that impede families coming in…. Less of a position of, ‘This is what you need, and I’m right because I’m the clinician,’ versus, “How do I understand where this family is coming from, and how do we then work together to really support the parents to see that their young person is struggling?’ So I think for me, it’s thinking ‘Okay, this is the model, and what are the culturally-responsive modifications so that it lands with families?’”
*Organizational setting*	
Need for case management interfering with FBT implementation	“I’ve had that situation happening with some of my DBT clients where there was like a high need of case management, and I got assigned a case manager that can hold that piece. I’m thinking that if that would have been the case, I would have had more time to address like FBT-related issues with the family and interventions, and I do not think of myself as a great case manager; there’s much more capable people. It would have served the family a lot more if they could have gotten that support.”
Difficulties team members were not aligned with FBT	“I was really mindful of the language that I used with mom [because of internalized mental health stigma in the family]. I tried not to talk a lot about the “eating disorder” but more about the need to gain weight or improve nutritional status, or eating behaviors. But that also got really tricky because then they would go see their pediatrician, who would tell them that he was doing great, and weight was great, and not to worry, and it would totally undermine our treatment because I was trying to intensify the concern around lack of eating, or weight loss, or lack of weight gain.”

## 4 Discussion

**Figure 2:**
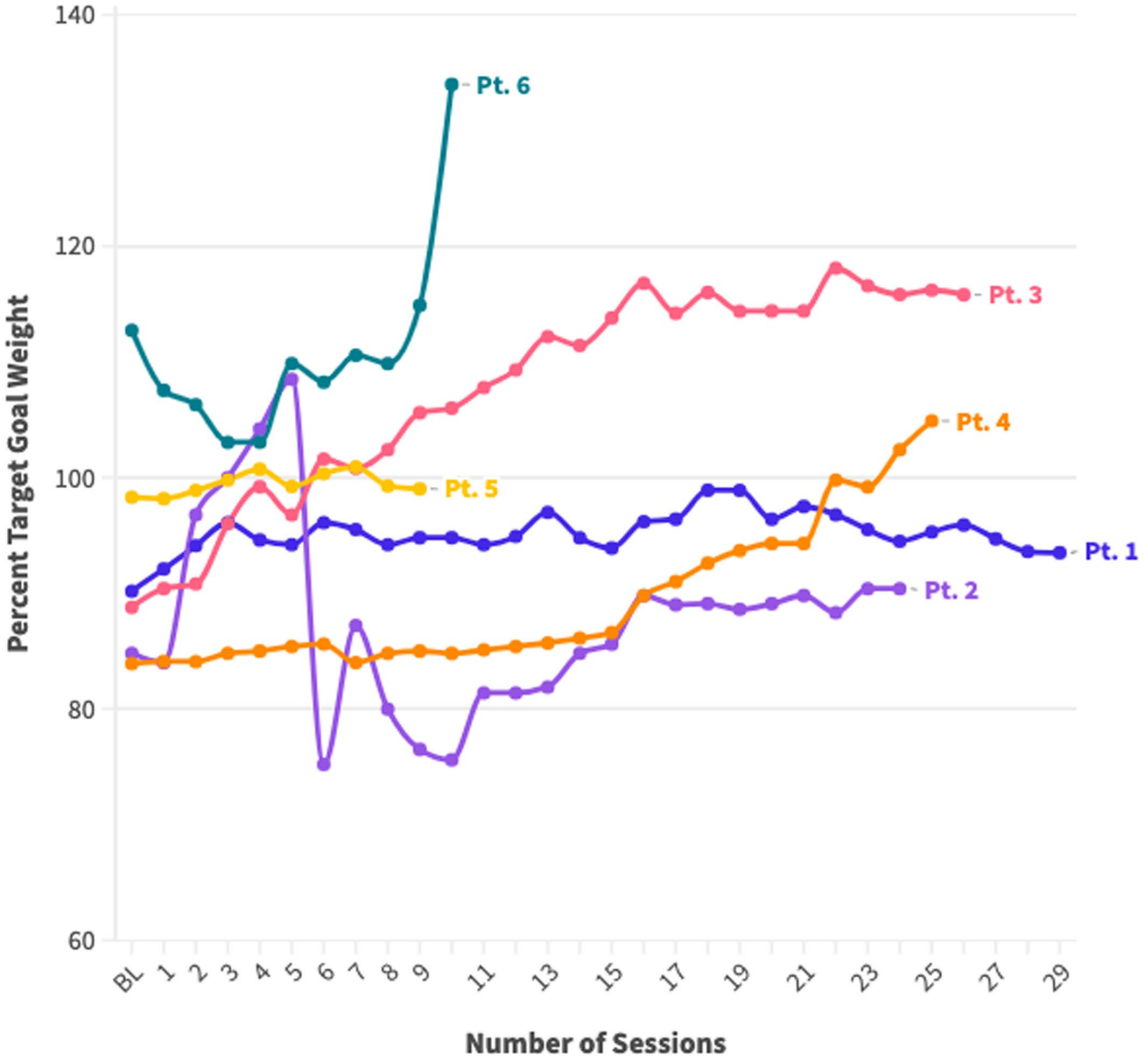
Changes in %BMI over FBT sessions.

FBT is currently the leading treatment for adolescents with AN and has been primarily studied in specialized eating disorder programs within academic medical centers, with little attention to how FBT might be delivered in community-based settings (Agras et al., 2014; Lock and Le Grange, 2015; Accurso et al., 2018). Given that EDs affect all demographic groups, it is critical that ED interventions be studied in more diverse populations, including historically marginalized groups, such as economically-disadvantaged families and racial and ethnic minorities, who are less likely to receive culturally appropriate, evidence-based treatments (Hayes et al., 2015; Halbeisen et al., 2022). Participants in this case series diverge from participants typically included in prior treatment trials (e.g., racially and ethnically diverse, bilingual, lower socioeconomic background). These mixed methods data suggest that an adaptable version of FBT for adolescents with anorexia nervosa is acceptable to patients, caregivers, and clinicians, with favorable patient outcomes, including weight restoration (Figure 2).

The qualitative results of this study also shed light on several important themes related to the characteristics of the FBT intervention and its implementation in various clinical settings. Cultural considerations emerged as a significant theme impacting FBT implementation. The clinicians in this study noted several cultural barriers that impeded successful implementation and highlighted how cultural adaptations of FBT may increase effectiveness and appropriateness across diverse populations, including paying special attention to family dynamics, religious practices, and gender roles when tailoring the treatment protocols. Clinicians also frequently referenced the importance of comprehensive psychoeducation while orienting the family to FBT. Study results are consistent with the adaptations outlined in Accurso and colleague’s protocol for adapting FBT in publicly-funded settings, such as placing additional emphasis on medical analogies to assist with externalization given the varying levels of health literacy (Accurso et al., 2021). Clinicians may also benefit from focusing on caregiver strengths and allowing space for discussions around caregiver roles in the contexts of systemic and linguistic barriers within systems of care (Accurso et al., 2021).

Socioeconomic considerations were another important theme that emerged from the qualitative data. Clinicians expressed initial concerns about implementing FBT in publicly-funded settings, particularly for Medicaid recipients. They recognized the challenges posed by limited resources and financial insecurity in these families. The structure of FBT was seen as potentially conflicting with the need for flexibility in addressing socioeconomic factors. Clinicians acknowledged the unrealistic nature of solely focusing on eating disorder symptoms when families faced urgent concerns related to financial stability, food insecurity, and housing. They recognized the privilege inherent in a narrow focus on the eating disorder and discussed the importance of addressing broader issues affecting families’ daily lives. The adaptations made in treatment included incorporating case management sessions to address food insecurity, assisting families in accessing resources, and providing additional support beyond the core components of FBT.

Logistical considerations, many of which were also related to socioeconomic factors, were also highlighted as a barrier to treatment implementation. Clinicians discussed challenges related to scheduling, transportation, and attendance of family members in sessions. Lack of transportation options, particularly for families without cars, presented a significant barrier to engagement. To overcome these challenges, clinicians explored alternative methods such as conducting sessions through telehealth video conferencing platforms. They also adapted the structure of sessions to accommodate the availability of family members, involving them at different points in the treatment process or providing take-home points to share with absent family members. In some cases, clinicians recognized the impracticality of involving family members in every session and explored modified approaches within the FBT framework.

Despite these challenges in implementation, clinicians generally held positive views of FBT and appreciated its alignment with a strengths-based perspective. They also believed in the effectiveness of FBT, appreciating its focus on eating behaviors and its ability to support families to feel less overwhelmed. They also acknowledged the flexibility of FBT in accommodating different family dynamics and cultural humility perspectives. This theme emphasizes the importance of FBT’s fit with the circumstances and presentations of patients within diverse clinical settings (Gorrell et al., 2019). The theme of complexity and learnability indicated that clinicians found FBT to be a comprehensible and well-defined treatment, although challenges in adapting the treatment to suit specific patient populations were mentioned including cultural beliefs, stigma and systemic barriers. Clinicians also emphasized the importance of collaboration between the mental health team and medical providers and highlighted challenges in coordinating care when working with non-specialized medical professionals.

Thematic saturation was reached with ten participating clinicians, but this study is limited by the fact that all participants were working within a single county, which has more local resources than many other counties in California (e.g., access to a local academic medical center specializing in inpatient and outpatient medical management of eating disorders). Generalizability may be limited, as other less resourced counties may face greater barriers to implementation without as much local infrastructure and ED expertise. The training model used in this study was resource-intensive (i.e., in-person training conducted over multiple days with weekly follow-up consultation), which is not a scalable approach (Accurso et al., 2021). Additionally, participants’ overall skill level may also not be generalizable to the rest of the country, with clinician participants being highly experienced in providing evidence-based treatments to diverse populations in community mental health settings. Their extensive experience likely contributed to their ability to make necessary cultural adaptations and address logistical concerns effectively. This level of expertise and comfort with making such adaptations may not be as prevalent among other community-based clinicians across the country, particularly in areas with fewer resources and less access to specialized training. Consequently, these clinicians might face greater difficulties in adapting FBT to meet the needs of their clients, potentially impacting the effectiveness and consistency of the treatment. Future research should consider these variations in clinician expertise and explore ways to enhance training programs to better prepare clinicians for the complexities of delivering FBT in diverse and under-resourced environments.

Case series findings also support the adaptability of FBT. The positive trends in engagement and outcomes among adolescents with AN, including target weight restoration and successful program completion for the majority. Notably, the participants in this case series differed from those in traditional treatment trials, being bilingual and coming from low socioeconomic backgrounds with parents as essential workers. Despite these challenges, the adaptable version of FBT demonstrated favorable outcomes, emphasizing the potential success of tailoring FBT to address the unique needs of diverse populations, including language barriers and socioeconomic factors. Some of the clinical adaptations employed may also enhance the quality of services for other low-income and ethnically, racially, and linguistically diverse groups.

The findings of this study align with existing literature highlighting how families from culturally diverse backgrounds can benefit from FBT when clinicians prioritize cultural competency. For example, Iguchi and colleagues described a series of adaptations made to FBT that were responsive to systemic and cultural barriers in Japan. The adaptations led to notable improvements, such as a reduced number of hospitalizations and a shorter time for patients achieve weight restoration (Iguchi et al., 2021). Similarly, another study in Denmark found positive outcomes in weight restoration and treatment completion when clinicians adapted FBT by spending more time addressing family system dilemmas throughout treatment (Bentz et al., 2021). These examples underscore the importance of culturally informed modifications to manualized treatments. By tailoring FBT to meet the specific cultural and systemic needs of diverse populations, clinicians can enhance the relevance and effectiveness of the protocol.

Overall, these findings provide valuable insights into the implementation of FBT in diverse and under-resourced clinical settings consistent with previous literature on clinical adaptations for multicultural patients. Understanding these themes can inform the development of strategies to enhance the successful implementation of FBT in diverse clinical contexts, taking into account the specific needs and challenges of patients and their families. Further research is needed to investigate the effectiveness of implementing these clinical adaptations as well as refinements to the adapted treatment model in “real world” contexts.

## Data availability statement

The data analyzed in this study are subject to the restrictions by UCSF’s IRB. These data are available upon reasonable request from the corresponding author, following authorization from the IRB and UCSF. Requests to access these datasets should be directed to Erin Accurso, erin.accurso@ucsf.edu.

## Ethics statement

The studies involving humans were approved by Institutional Review Board (IRB) at the University of California, San Francisco (UCSF). The studies were conducted in accordance with the local legislation and institutional requirements. The participants provided their written informed consent to participate in this study.

## Author contributions

RB: Formal analysis, Writing – original draft, Writing – review & editing. PC: Formal analysis, Writing – review & editing. JL: Conceptualization, Funding acquisition, Methodology, Writing – review & editing. JG: Conceptualization, Funding acquisition, Methodology, Writing - review and editing. EA: Conceptualization, Data curation, Formal analysis, Funding acquisition, Investigation, Methodology, Project administration, Resources, Software, Supervision, Validation, Writing – review & editing.
